# Production and Immunogenicity of Soluble Plant-Produced HIV-1 Subtype C Envelope gp140 Immunogens

**DOI:** 10.3389/fpls.2019.01378

**Published:** 2019-10-30

**Authors:** Emmanuel Margolin, Rosamund Chapman, Ann E. Meyers, Michiel T. van Diepen, Phindile Ximba, Tandile Hermanus, Carol Crowther, Brandon Weber, Lynn Morris, Anna-Lise Williamson, Edward P. Rybicki

**Affiliations:** ^1^Division of Medical Virology, Department of Pathology, Faculty of Health Sciences, University of Cape Town, Cape Town, South Africa; ^2^Biopharming Research Unit, Department of Molecular and Cell Biology, University of Cape Town, Cape Town, South Africa; ^3^Institute of Infectious Disease and Molecular Medicine, Faculty of Health Sciences, University of Cape Town, Cape Town, South Africa; ^4^National Institute for Communicable Diseases of the National Health Laboratory Service, Sandringham, South Africa; ^5^Structural Biology Research Unit, Division of Medical Biochemistry, Department of Integrative Biomedical Sciences, University of Cape Town, Cape Town, South Africa; ^6^Faculty of Health Sciences, University of Witwatersrand, Johannesburg, South Africa

**Keywords:** HIV, glycoprotein, plants, immunogenicity, modified vaccinia Ankara

## Abstract

The development of effective vaccines is urgently needed to curb the spread of human immunodeficiency virus type 1 (HIV-1). A major focal point of current HIV vaccine research is the production of soluble envelope (Env) glycoproteins which reproduce the structure of the native gp160 trimer. These antigens are produced in mammalian cells, which requires a sophisticated infrastructure for manufacture that is mostly absent in developing countries. The production of recombinant proteins in plants is an attractive alternative for the potentially cheap and scalable production of vaccine antigens, especially for developing countries. In this study, we developed a transient expression system in *Nicotiana benthamiana* for the production of soluble HIV Env gp140 antigens based on two rationally selected virus isolates (CAP256 SU and Du151). The scalability of the platform was demonstrated and both affinity and size exclusion chromatography (SEC) were explored for recovery of the recombinant antigens. Rabbits immunized with lectin affinity-purified antigens developed high titres of binding antibodies, including against the V1V2 loop region, and neutralizing antibodies against Tier 1 viruses. The removal of aggregated Env species by gel filtration resulted in the elicitation of superior binding and neutralizing antibodies. Furthermore, a heterologous prime-boost regimen employing a recombinant modified vaccinia Ankara (rMVA) vaccine, followed by boosts with the SEC-purified protein, significantly improved the immunogenicity. To our knowledge, this is the first study to assess the immunogenicity of a near-full length plant-derived Env vaccine immunogen.

## Introduction

Prophylactic vaccines are urgently needed to combat HIV-1, particularly in Sub-Saharan Africa which remains disproportionately affected by the pandemic and accounts for the majority of new infections and AIDS-related deaths (Shao and Williamson 2012). It is estimated that ~1.8 million new infections and ~1 million deaths from AIDS-related illnesses occurred in 2016 alone (www.unaids.org). Alarmingly, Sub-Saharan Africa accounted for 64% of new infections during this period. Despite the impact of improved access to antiretroviral therapy on disease incidence, new infections continue to occur; consequently, even a partially effective vaccine is expected to have a large impact on the epidemic (Medlock et al., 2017).

Although none of the candidate vaccines evaluated in clinical trials have achieved the level of efficacy required for licensure, recent insights into the development of broadly cross-neutralizing antibodies during natural infection and advances in the rational design of immunogens have renewed interest in the development of prophylactic vaccines (Stephenson et al., 2016; McCoy and McKnight 2017; Sanders and Moore, 2017). Additionally, the RV144 trial in Thailand showed that vaccine-mediated protection against HIV acquisition is possible, and subsequent analyses have given insight into the kind of responses that a vaccine may need to elicit to achieve protection (Rerks-Ngarm et al., 2009, Haynes et al., 2012; Liao et al., 2013). It is also evident that non-neutralizing antibodies against Env contribute to protection against HIV infection by means of Fc-mediated effector functions (Rerks-Ngarm et al., 2009; Haynes et al., 2012; Barouch et al., 2015; Alter and Barouch 2018).

During natural infection with HIV, a subset of infected individuals develop broadly cross-neutralizing antibody responses against the Env gp160 glycoprotein (Li et al., 2007; Doria-Rose et al., 2009; Sather et al., 2009; Simek et al., 2009; Doria-Rose et al., 2010; Euler et al., 2010; Gray et al., 2011; Hraber et al., 2014; Landais et al., 2016). However, these responses typically occur late during infection and do not usually confer any obvious clinical benefit (Blish et al., 2008; Euler et al., 2010; Gray et al., 2011). The passive transfer of Env-specific neutralizing monoclonal antibodies can protect against viral challenge in non-human primates, suggesting that they would be able to prevent human infection if present at the sites of exposure (Mascola et al., 1999; Parren et al., 2001; Hessell et al., 2009; Rosenberg et al., 2013; Shingai et al., 2014). Many of these broadly neutralizing antibodies preferentially recognize Env epitopes in the context of trimers, as the epitope may span more than one of the protomers of the spike (Walker et al., 2009; Walker et al., 2011; Julien et al., 2013; Doria-Rose et al., 2014; Sok et al., 2014; Longo et al., 2016). Thus, a major focal point of current HIV vaccine research is the production of rationally designed Env trimers which resemble the native, virion-bound glycoprotein spikes (Sanders and Moore, 2017).

These trimer mimetics are designed to both occlude immunodominant, non-neutralizing epitopes that are inaccessible in the native trimer, and preferentially present epitopes targeted by broadly neutralizing antibodies (Sanders et al., 2013). However, the production of these antigens in mammalian cell culture is expensive, and the requisite infrastructure to produce at any scale higher than laboratory-based cultures is largely absent in developing countries. Plant-based expression of heterologous proteins is an attractive alternative to cell culture-based techniques, especially for resource-limited regions, due to the potential for greatly reduced production costs, rapid scalability and less stringent infrastructure requirements (Hefferon 2013; Ma et al., 2013). HIV vaccine implementation will also require unprecedented scalability and the potential need for annually repeated immunizations, as already occurs with seasonal influenza vaccines, could eclipse all available manufacturing capacity (Mortimer et al., 2012). Recently, high yields and promising immunogenicity of plant-produced influenza virus haemagglutinin-derived antigens have been reported, several of which have advanced into clinical trials (D’Aoust et al., 2008; Shoji et al., 2008; Shoji et al., 2009a; Shoji et al., 2009b; Bosch and Schots 2010; Landry et al., 2010; Madhun et al., 2011; Chichester et al., 2012; Shoji et al., 2012; Cummings et al., 2014). Medicago Inc (USA) has also demonstrated the scalability of plant-based expression by producing 10 million doses of fully formulated influenza H1N1pdm vaccines in one month (Yusibov et al., 2015).

Given the recent successes of the expression of influenza immunogens in plants, and the structural similarities between the influenza and HIV glycoproteins, it is feasible that plants may be able to produce suitable Env glycoproteins (D’Aoust et al., 2008; Karlsson Hedestam et al., 2008; Shoji et al., 2008). A number of groups have successfully expressed variable regions of gp120 or portions of gp41, as fusions with either plant virus capsid proteins or using cholera toxin B as a carrier (Yusibov et al., 1997; Durrani et al., 1998; Marusic et al., 2001; Kim et al., 2004). Although these vaccine candidates were immunogenic, they do not faithfully reproduce the conformation of these regions. More recently, Gag-based VLPs presenting the membrane-proximal external region (MPER) of gp41 were produced in *N. benthamiana* plants (Kessans et al., 2016).

The most promising study to date was conducted by Rosenberg and colleagues, who expressed a truncated, soluble Env protein in *N. benthamiana* plants—but as a reagent for characterization of plant-made antibodies, rather than as a vaccine candidate. The protein was a soluble gp140—with the gp41 truncated by removal of both the cytoplasmic and transmembrane domains—that also had the cleavage site, fusion peptide, and immunodominant region of gp41(∆CFI) removed (Rosenberg et al., 2013). It reacted with several prototype monoclonal antibodies, including 2G12 which recognizes a glycan-dependent epitope on the outer domain of Env (Rosenberg et al., 2013). However, its immunogenicity was not reported and it remains unclear if the antigen was trimeric. A similarly modified consensus Env (Con-S ∆CFI) was expressed as a fusion with the influenza haemagglutinin transmembrane and cytoplasmic domains (D’Aoust et al., 2011). While expression of a SIV gp130 protein was described in transgenic maize seed, once again no immunogenicity was reported (Horn et al., 2003).

It has been shown that proteolytic cleavage at the interface of the gp120 and gp41 subunits is important for the proper native conformation (Ringe et al., 2013). Recently, however, native-like soluble Env trimer mimetics were produced, in the absence of cleavage, by substituting the cleavage motif for a flexible linker peptide (Georgiev et al., 2015; Sharma et al., 2015). This approach is attractive for heterologous expression systems, such as plants, where endogenous furin activity is lacking (Wilbers et al., 2016). Our group has been investigating the production of cleavage-independent HIV Env gp140 antigens in mammalian cells (van Diepen et al., 2018) and their suitability as a booster vaccine for prior priming by DNA and/or modified vaccinia Ankara vaccines encoding modified Gag and a gp150 Env (van Diepen et al., 2018). In this study, we report the development of an *Agrobacterium*-mediated transient expression system for the production of cognate soluble HIV-1 subtype C gp140 antigens in *N. benthamiana* plants, and immunological studies of these proteins in rabbits.

## Materials and Methods

### Antigen Design

**Figure 1:**
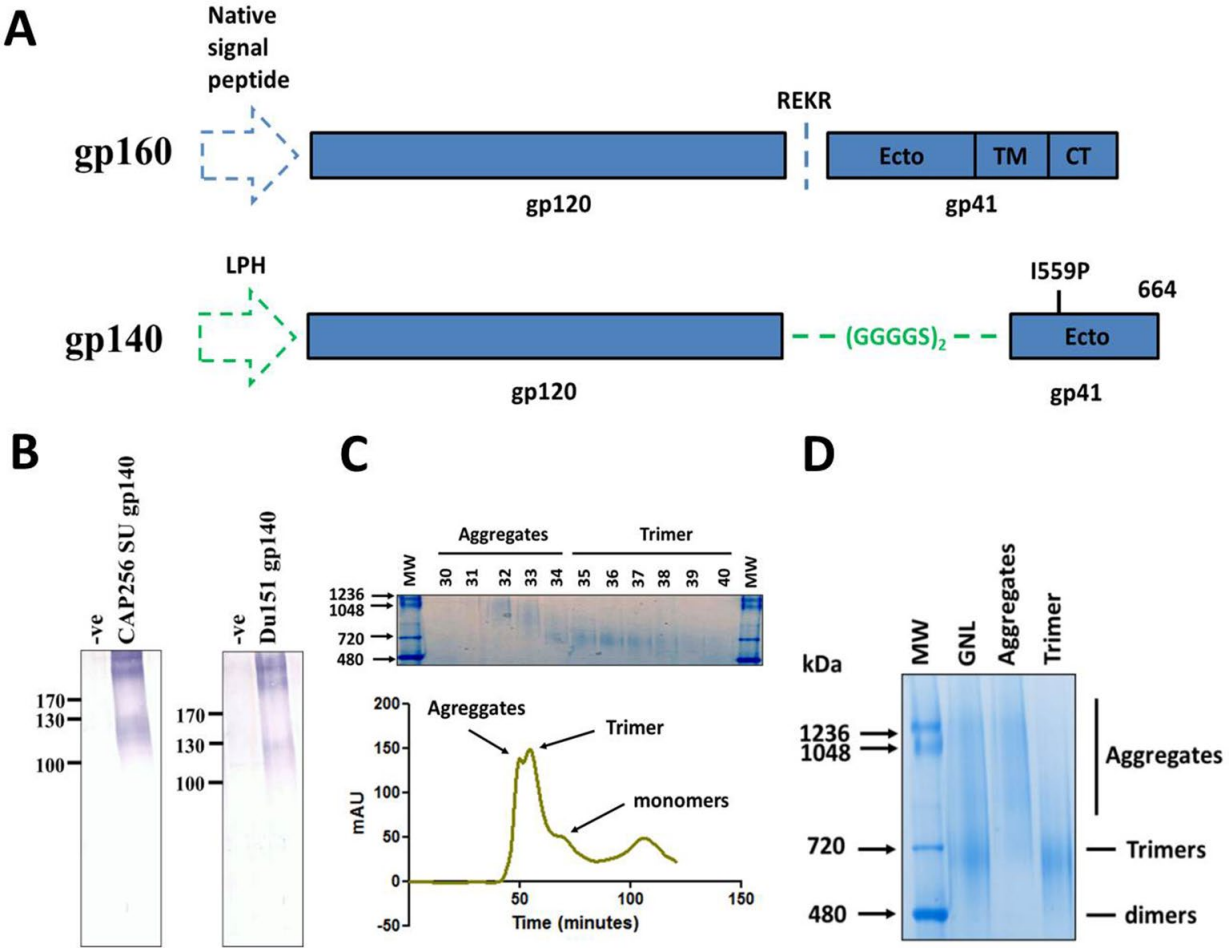
Design, expression and purification of HIV Env gp140 antigens. **(A)** Schematic of the coding sequence for the native HIV-1 gp160 gene (top) and the soluble gp140 antigen (bottom). The gp120 and gp41 portions of the proteins are delineated with either the native cleavage sequence (REKR) or flexible linker peptide (GGGGS)_2_ at the interface of the two subunits. The location of the I559P helix breaking mutation and amino acid residue 664, where the coding sequence was terminated, is reflected for the gp140 antigen. The native and LPH (leader peptide heavy chain) signal sequences are shown by the blue and green dashed arrows respectively. (Ecto = ectodomain, TM = transmembrane, CT = cytoplasmic tail). **(B)** Western blot confirming expression of CAP256 SU gp140 (left) and Du151 gp140 (right) in crude leaf homogenate, 5 days post infiltration. Blots were probed with polyclonal goat anti-HIV-1 gp120 antibody. The negative controls comprise of leaf tissue agroinfiltrated with recombinant *A. tumefaciens* AGL1 that has been transformed with the empty pEAQ-HT expression vector (-ve). Both CAP256 SU Env and Du151 Env contain 29 putative N-glycosylation sites. **(C)** Superdex 200 Hiload 16/600 elution profile of fractionated CAP256 SU Env species following affinity chromatography. The identities of the different protein species are indicated on the profile. A Coomassie-stained BN-PAGE gel of the fractionated material is shown above the graph indicating the fractions that comprise the aggregates and presumed trimer peaks. **(D)** Coomassie stained BN-PAGE gel containing the pooled and concentrated CAP256 SU Env samples corresponding to peaks in the elution profile peaks. An aliquot of the affinity-purified protein (GNL), prior to SEC, was resolved alongside aggregate and trimer samples for comparison. The trimers indicated in both panels **(C)** and **(D)** are based on the SEC elution profiles and BN-PAGE migration of other HIV-1 gp140 antigens in the published literature.

Soluble cleavage-independent HIV Env gp140 antigens were designed as described by Sharma et al., 2015 (**Figure 1**), obviating the need for furin-mediated proteolytic cleavage which does not occur naturally *in planta* (Sharma et al., 2015, Wilbers et al., 2016). The native HIV Env cleavage site was replaced with a 10 amino acid flexible linker comprising of 2 repeats of the glycine-serine based (GGGGS) motif. The isoleucine at residue 559 in the N-terminal heptad repeat of gp41 was mutated to a proline and the coding sequence prematurely terminated by the introduction of a stop codon after amino acid residue 664. The coding sequence of the full length Env from the HIV CAP256 SU virus (clone 256.2.06.c7) was provided by Dr. Penny Moore (Centre for HIV and STIs, National Institute for Communicable Diseases, Johannesburg) and Daniel Sheward (HIV Diversity and Pathogenesis Research Group, University of Cape Town). The HIV-1 Du151 Env sequence was retrieved from GenBank (Accession number AF544008.1). The gene coding sequences were synthesized by GenScript, after optimization, to reflect the preferred human codon usage and the addition of synthetic Age1 and Xho1 restriction sites at the 5’ and 3’ terminal ends of the genes, respectively. A synthetic Not1 site was included prior to the stop codon resulting in a run of three alanine residues at the C terminal end of the protein. Lastly, the native signal sequence was replaced with the murine mAB24 heavy chain-derived LPH (leader peptide heavy chain) signal peptide, to direct translocation of the recombinant protein through the plant secretory pathway.

### Assembly of pEAQ-HT Expression Vectors Encoding HIV-1 Env Antigens

The gp120 and gp41 regions of the Env antigens were synthesized separately and assembled in pUC57, before they were subcloned into pEAQ-HT (Margolin, 2017). The gp140 coding sequences were excised from their respective pUC57 vector backbones using Age1 and Xho1. Similarly, the pEAQ-*HT* expression vector was digested with Age1 and Xho1 to generate compatible sticky ends for cloning. The genetic integrity of the final clones was verified by restriction analysis and sequencing across the cloning junctions with vector-specific primers (5’ TTCTTCTTCTTGCTGATTGG3’ and 5’ CACAGAAAACCGCTCACC 3’, respectively) which bind to the pEAQ-*HT* vector on either side of the multiple cloning site. The expression constructs were electroporated into *A. tumefaciens* AGL1 as previously described (Maclean et al., 2007). Transformants were selected for on Luria Bertani agar supplemented with 50 μg/ml kanamycin and 25 μg/ml carbenicillin. Putative transformants were screened by PCR of isolated plasmid DNA using the same primer pair.

### Propagation of *N. benthamiana* Biomass

*N. benthamiana* seeds were germinated in flat trays filled with soil and incubated at 25°C (55% humidity), under a regulated 16-h light/8-h dark photocycle. After 3 weeks, individual seedlings were transplanted into pots containing a 2:1 mixture of peat to vermiculite. Plants were infiltrated with recombinant *A. tumefaciens* strains at 6–8 weeks and then returned to the greenhouse, under the same environmental conditions, for the duration of the experimental procedure.

### Transient Expression of Recombinant HIV-1 Env Antigens in *N. benthamiana* Leaves

Glycerol stocks of recombinant *A. tumefaciens* AGL1 were revived in 10 ml LB media, supplemented with 25 µg/ml carbenicillin (Sigma-Aldrich) and 50 µg/ml kanamycin (Sigma-Aldrich). The cultures were sequentially scaled up to an appropriate volume in LB base medium [2.5 g/l tryptone, 12.5 g/l Yeast extract, 5 g/l NaCl, 10 mM MES (pH 5.6)], with 20 µM acetosyringone supplemented during the final culture step. The bacterial suspension was then adjusted to an OD_600_ of 1.0, using freshly prepared resuspension medium (10 mM MgCl_2_, 10 mM MES [pH5.6], 200 µM acetosyringone). Whole plants were submerged, upside down, in a beaker of the bacterial culture placed inside a vacuum chamber. A vacuum of -80 kPa was applied to the chamber and the procedure repeated 2–3 times to ensure complete infiltration of the leaves. The agroinfiltrated plants were then returned to the greenhouse and incubated under the same environmental conditions until harvest.

### Small Scale Extraction of Crude Soluble Protein

Clippings were harvested from agroinfiltrated leaves and finely ground in liquid nitrogen. The leaf material was resuspended in PBS [Lonza] (50 μl/clipping), supplemented with cOmplete^™^ EDTA-free protease inhibitor as per the manufacturers instructions (Roche), and incubated at 4°C for 1 h, with shaking. The plant slurries were clarified by centrifugation at 14,000 rpm, for 15 min, and the supernatant stored at -20°C.

### Affinity Purification of Recombinant HIV-1 Env Glycoproteins

The aerial parts of the plants were harvested 5 days post agroinfiltration and homogenized in two buffer volumes of PBS (Lonza), supplemented with cOmplete^™^ EDTA-free protease inhibitor (Roche). The crude homogenate was incubated for 1 h, at 4°C, with shaking and then filtered through four layers of Miracloth^™^ (Merck). The crude plant sap was then clarified by sequential centrifugation steps; twice at 15,344 × *g* for 20 min and then again at 17,000 × *g* for 20 min. The supernatant was vacuum-filtered through a 0.45 µM Stericup-GP device (Merck Millipore) and applied to a *Galanthuis nivalis* lectin (GNL) column (Sigma) with a 0.5–1 ml/min flow rate. The column was sequentially washed with 100 ml of 0.5 M NaCl and then 100 ml of PBS (Lonza). The proteins were eluted in 1 M methyl α-D-manno-pyranoside (MMP) (Sigma), buffer exchanged into PBS and then concentrated using a Vivaspin Protein Concentrator with a 30 kDa cut-off (GE Healthcare). The purified proteins were quantified using the DC^™^ Protein Assay (Bio-Rad).

### SEC Purification of HIV Env Glycoprotein Antigens

Following elution from the GNL affinity resin, the recombinant protein was concentrated and buffer- exchanged into 5 ml of PBS [pH 7.4] (Lonza). The purified protein was then size-fractionated using a Superdex 200 HiLoad 16/600 column (GE Healthcare). Fractions corresponding to the chromatogram peaks were analyzed by non-denaturing BN-PAGE followed by Coomassie staining to confirm their oligomeric identity. The desired fractions were pooled and stored at -80°C for immunogenicity studies.

### Electrophoretic Resolution of Proteins and Western Blotting

Protein samples were resolved under denaturing conditions by 10% SDS-PAGE. Alternately, samples were resolved in their native state using NativePAGE^™^ Novex^®^ 3–12% Bis-Tris Gels in accordance with the manufacturer’s instructions. Following electrophoresis, proteins were either electrophoretically transferred onto Immun-blot^®^ PVDF Membrane (Bio-Rad) or stained with Bio-Safe^™^ Coomassie stain (Bio-Rad). PVDF membranes were blocked for 2 h in 2% BSA and Env protein detected with a 1:1,000 dilution of goat anti-HIV-1 gp120 antibody (AbD Serotec). In turn, the primary antibody was detected with 1:10,000 dilution of GT34 anti-sheep/goat secondary antibody (Sigma-Aldrich). Western blots were developed with 5 ml BCIP/NBT substrate (KPL) for 30 min.

### Protein Identity Determination by Liquid Chromatography-Mass Spectrometry (LC-MS)

The identities of Coomassie-stained protein bands were independently determined by the Centre for Proteomic and Genomic Research (CPGR, Cape Town). Protein bands were recovered from the gel and fragmented by trypsin digestion, alongside a BSA reference standard. The resulting peptide solution was separated using the Dionex Ultimate 3,000 nano-HPLC system (ThermoFischer Scientific, USA) and then analyzed using a Q Exactive^™^ Hybrid Quadrupole-Orbitrap Mass Spectrometer (ThermoFischer Scientific, USA). The spectra generated by LC-MS were analyzed with Byonic Software (Protein Metrics USA) using publically available sequences retrieved from UniProt (www.uniprot.org). Samples were interrogated against a merged database comprised of *N. benthamiana*, *N. tabacum*, *Agrobacterium*, and HIV proteomes.

### Rabbit Immunizations

Rabbit immunizations and blood sampling was conducted at the University of Cape Town Research Animal Facility, in accordance with the guidelines and approval of a Faculty Animal Ethics Committee (AEC 014-30) and at the Animal Unit of the University of Stellenbosch in accordance with the guidelines and approval of the UCT Committee (AEC 015-05). Three-month-old New Zealand White rabbits (> 2 kg) were immunized with 50 µg of recombinant protein suspended in Alhydrogel^®^ Adjuvant 2% (Invivogen) at a concentration of 1:1 (antigen: adjuvant), determined to be the optimal adjuvant in other work with mammalian cell-made HIV-1 gp140 in our lab (van Diepen et al., 2018). Groups of five rabbits were immunized intramuscularly into the quadriceps muscle of the hind leg at weeks 0, 4, 12, and 20. Animals in the last group were inoculated with 1 × 10^8^ pfu rMVA at weeks 0 and 4, followed by immunization with 50 μg of the adjuvanted SEC-purified protein at weeks 12 and 20. Blood was drawn at weeks 0, 4, 8, 12, 14, 16, 20, 22, and 24 weeks for analysis. The experiment was terminated after 24 weeks.

### Quantification of Serum Antibody Binding Titres

The levels of serum binding antibodies were quantified by ELISA using gp140 produced in HEK293 cells as a coating antigen. The mammalian cell-derived protein was purified the same way as plant-produced protein. SEC-purified protein was used as a coating antigen for animals immunized with SEC-purified gp140, and lectin affinity purified antigen for the cognate plant product where SEC was not performed after affinity purification. The assay was conducted as previously described (van Diepen et al., 2018; van Diepen et al., 2019).

Serum-binding antibodies to the autologous V1V2 Env region were quantified by ELISA using a protein scaffold provided by Professor Penny Moore (Senior Medical Scientist, Centre for HIV and STIs, National Institute for Communicable Diseases, Johannesburg). Ninety-six-well Maxisorb^®^ microtitre plates (NunC) were coated overnight with 450 ng of purified scaffold and the assay conducted as before. Binding antibody levels were determined as a fold-dilution derived from the fitted four-point linear regression curve using a threshold of the minimum + standard error of the minimum for each time point. Env-binding antibodies and V1V2 binding antibodies were both quantified 4 weeks after the 3^rd^ (week 16) and 4^th^ (week 24) immunization. Binding antibodies were quantified using SEC-purified CAP256 SU gp140 protein that was purified from HEK293 cells.

### Serum Neutralization of Env-Pseudotyped Viruses

The serum from immunized animals was assessed for HIV-1 pseudovirus neutralization at the National Institute for Communicable Diseases (Sandringham, Johannesburg). Neutralizing activity was measured in TZM-bl cells as the ability of serum to reduce luciferase reporter gene expression after a single round of infection with replication-incompetent Env-pseudotype viruses (Montefiori, 2005). Two highly sensitive Tier 1A viruses (MW965.26, MN.3), 2 moderately sensitive Tier 1B (6,644, 1,107,356) viruses and the Tier 2 autologous viruses (CAP256 SU and Du151.2) which are more resistant to neutralization were tested. Env-pseudotyped viruses were generated by transient co-transfection of HEK293T cells with pSG3∆Env, containing an HIV genome with a defective Env gene, and a complementary plasmid encoding the Env gene of interest (pcDNA 3.1D/V5-His-TOPO-Env). Serially diluted sera were incubated with pseudovirions and then overlaid on TZM-bl cells seeded in a 96-well flat-bottomed plate. The plates were incubated for 48 h before lysing the cells and assaying the lysate for luciferase activity. The ID_50_ was calculated as the reciprocal dilution required for a 50% reduction in relative luciferase units. Murine leukaemia virus (MuLV) was included as a negative control. Sera with no neutralizing activity were assigned an arbitrary value of 19 to allow for statistical analysis.

### Statistical Analysis

All statistical analyses were conducted using GraphPad Prism 5. Statistical comparisons between groups over time were determined using a two-way Anova test. Comparisons between two groups at a single time point were performed using a two-tailed Mann-Whitney unpaired test. In both cases, a P-value below 0.05 was considered to indicate a significant difference.

## Results

### Selection of HIV Env

The vaccine antigens used were based on two rationally selected HIV-1 subtype C Env sequences. The first was the superinfecting virus from participant CAP256 in the CAPRISA 002 Acute Infection Cohort (CAP256 SU), who developed broadly cross-neutralizing antibodies targeting the V1V2 loop (Moore et al., 2011; Moore et al., 2013; Doria-Rose et al., 2014). The CAP256 SU Env glycoprotein also has documented sensitivity to many prototype broadly neutralizing antibodies (Moore et al., 2011; Doria-Rose et al., 2014; Bhiman et al., 2015). The second was HIV Du151 Env, isolated in 1998 from an individual within the first 2 months of infection. It was selected as the vaccine strain by the South African AIDS Vaccine Initiative (SAAVI) in 2003 due to its close similarity to a South African subtype C consensus sequence (Williamson et al., 2003).

### Transient Expression of HIV-1 gp140 Glycoprotein Mimetics *In Planta*

Soluble gp140 antigens, reflecting the preferred human codon usage, were designed from the CAP256 SU and Du151 Env genes (**Figure 1A**). This codon usage was previously reported to result in higher levels of expression for the analogous influenza HA glycoprotein in plants (Mortimer et al., 2012), as well as for other antigens tested in the Biopharming Research Unit (human and bovine papillomavirus L1 proteins, pers. Comm) (Maclean et al., 2007). *N. benthamiana* plants were vacuum infiltrated with recombinant *A. tumefaciens* strains encoding the HIV-1 gp140 antigens from the pEAQ-*HT* expression plasmid. Expression of both proteins induced severe pathology in infiltrated plants, although the effect was more pronounced for plants expressing Du151 gp140, which displayed marked necrosis by 5 days post infiltration (dpi) (**Figure S1**). Accumulation of the recombinant antigens was monitored by western blotting of SDS-PAGE-separated crude leaf homogenate for 9 days. Expression of both antigens was detectable by 3 dpi and expression levels peaked after 5 days (data not shown). Western blots showed a product just below the 130 kDa molecular weight marker, as well as higher order molecular weight aggregates (>245 kDa) that were poorly resolved by SDS-PAGE (**Figure 1B**). There was no obvious improvement in the resolution of these aggregates when protein was extracted in buffers with different pH values, with detergent, or with an inhibitor of oxidation (data not shown). This is slightly smaller than the expected molecular weight of 140 kDa.

### Purification of Recombinant HIV-1 Env gp140

Protein production was scaled up by increasing the number of plants infiltrated, and the antigens were purified by lectin affinity chromatography. The mean recovery of the CAP256 SU and Du151 antigens was 6.2 mg/kg and 4.9 mg/kg of plant biomass, respectively (*n* = 3 independent infiltrations and purifications). Although the CAP256 SU gp140 demonstrated a trend towards higher expression, this was not statistically significant (unpaired *t* test, p > 0.05). Liquid chromatography mass spectrometry (LC-MS) analysis of Coomassie-stained bands following SDS-PAGE verified the purification of HIV Env and that the unresolved products were also Env. In addition, low levels of endogenous plant proteins were evident below the 80 kDa molecular weight marker (**Figure S2** and **Table S1**). The major contaminant of the purified CAP256 SU gp140 antigen was a homologue of *Agrobacterium fabrum* chaperone protein DNA K, although the coverage was poor, and this was most likely a region conserved amongst *Agrobacterium* strains. For Du151 gp140 the main contaminant was the tobacco luminal-binding protein 5, a homologue of the endoplasmic reticulum chaperone BiP.

We also tested a subsequent SEC step to remove plant protein contaminants and aggregated Env species from the purified CAP256 SU gp140 antigen. This protein was specifically selected for SEC due to its trend towards higher expression levels *in planta* compared to that of Du151 Env and the strong rationale underlying its selection for development as a vaccine immunogen. Size fractionation yielded two closely overlapping peaks followed by a small shoulder and another small peak (**Figure 1C**). Coomassie staining of pooled SEC peaks yielded products consistent with the sizes expected for aggregates (>720 kDa) and trimers (~720 kDa) following their resolution by BN-PAGE (Sharma et al., 2015) (**Figure 1D**). The small shoulder that eluted after the putative trimeric fraction was below the threshold of detection by Coomassie staining but is presumed to be monomeric protein. The identity of the small peak that eluted later is unknown. This approach resulted in the successful removal of the majority of contaminating aggregates and monomers, and the recovery of putative trimeric protein (**Figure 1D**). The yield of purified SEC-CAP256 SU gp140 protein was relatively low at ~1.94 mg recovered per kg of biomass.

### Autologous Serum-Binding Antibodies

**Figure 2:**
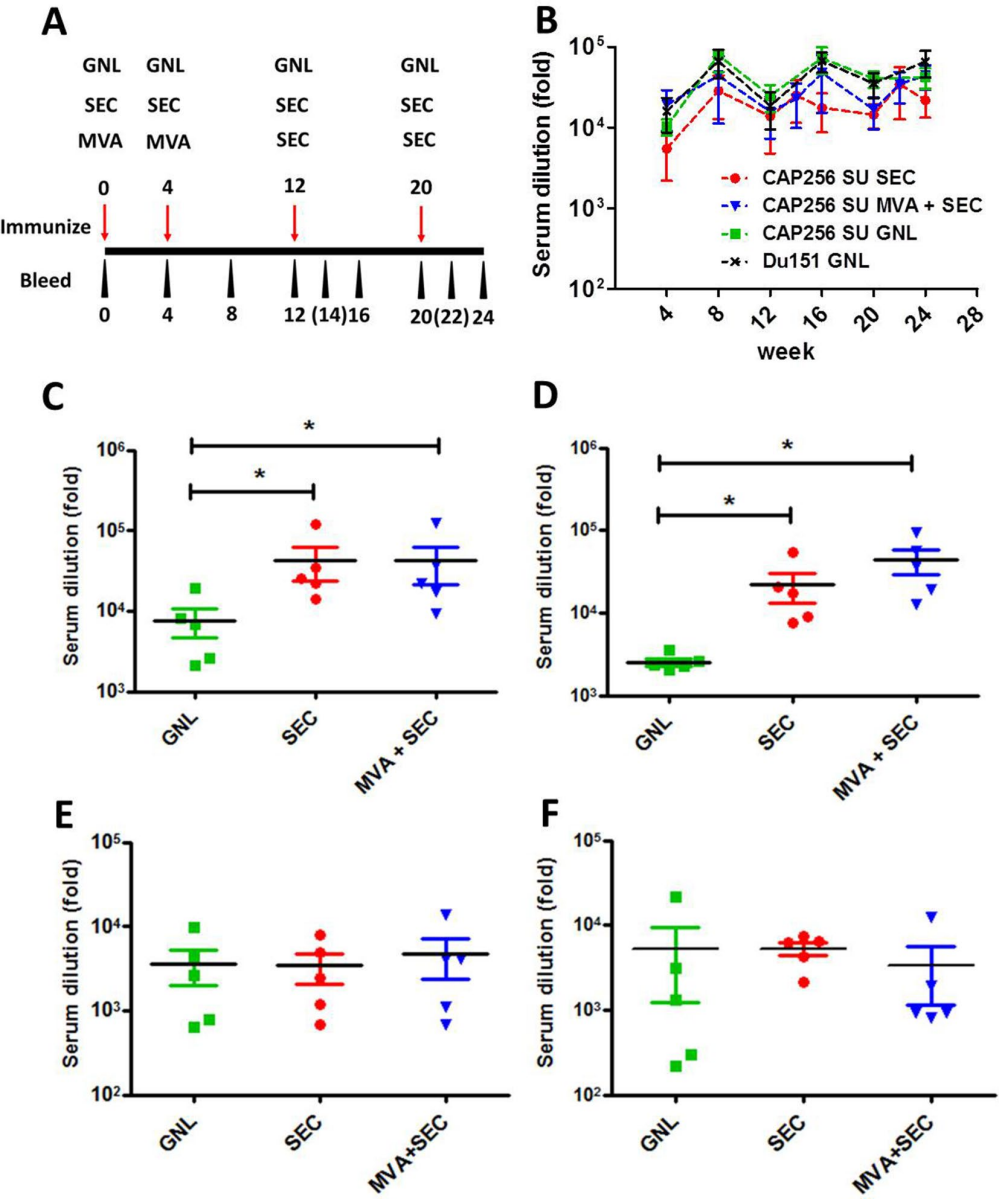
Immunogenicity of the different vaccination regimens in rabbits. **(A)** Immunization schematic indicating the timing of immunizations and bleeds. Time points in brackets indicate blood draws which were taken for the group immunized with the SEC-purified protein only. **(B)** Serum binding antibodies elicited over the course of the immunization regimen to the matched antigen, purified from mammalian cells, that was used to immunize animals. The levels of serum binding antibody titres elicited by the different vaccination regimens of CAP256 SU Env were compared after the 3^rd^ **(C)** and 4^th^ **(D)** immunizations. Similarly, autologous V1V2 binding antibodies were quantified after the 3^rd^ **(E)** and 4^th^ **(F)** inoculation. Statistical comparisons between groups were performed using a two-tailed, unpaired Mann-Whitney test (*p < 0.05). The levels of binding antibodies are indicated as a fold-dilution derived from the fitted four-point linear regression curve using a threshold of the minimum + standard error of the minimum for each time point.

The immunogenicity of the affinity-purified and SEC-purified antigens was tested by immunizing rabbits four times with 50 µg of protein, formulated in Alhydrogel^®^ adjuvant, at weeks 0, 4, 12, and 20. An additional group was included where animals were primed twice with rMVA encoding a subtype C mosaic Gag antigen (Jongwe et al., 2016) and a membrane-bound gp150 protein matched to our gp140 but truncated at amino acid 730 to remove most of the cytoplasmic domain (van Diepen et al., 2019), followed by two boosts with the SEC-purified plant-made Env (**Figure 2A**). The mosaic Gag was previously observed to form virus-like particles in cells transfected with DNA or infected with recombinant MVA encoding the protein. Furthermore, the antigen was highly immunogenic in both vaccine modalities (Jongwe et al., 2016; Chapman et al., 2017). Addition of gp150 to mosaic Gag DNA or MVA vaccines resulted in inclusion of Env in these Gag VLPs (van Diepen et al., 2019).

The vaccines were well tolerated and all groups of immunized animals rapidly developed binding antibodies which were detectable even after a single immunization (by week 4) (**Figure 2B**). Increased serum binding antibodies were observed over the course of the 24-week experiment, although no statistical difference was observed between groups over time or between individual time points (p > 0.05, two-way Anova).

The levels of SEC-purified Env-binding antibodies elicited by the different vaccination regimens were also quantified after the 3^rd^ and 4^th^ immunization as this probably represents a more authentic and vaccine-relevant form of the protein. Both groups of animals immunized with the SEC-purified protein had significantly higher levels of binding antibodies than animals immunized with the affinity-purified protein (P < 0.05, two-tailed Mann-Whitney unpaired test) (**Figures 2C, D**). No significant difference in binding antibody levels was observed between animals immunized with only SEC-purified protein and animals from the MVA prime-protein boost group.

The levels of V1V2 antibodies elicited by the CAP256 SU-derived vaccines were also quantified as the RV144 trial reported that reduced risk of HIV-1 acquisition was correlated with binding antibodies to this region (Haynes et al., 2012). Encouragingly, all vaccination regimens elicited autologous V1V2 scaffold binding antibodies which were detected at weeks 16 and 24 (**Figures 2E, F**). No statistically significant differences were observed between groups.

### Serum Neutralizing Antibodies Against Env-Pseudotyped Virions

**Figure 3:**
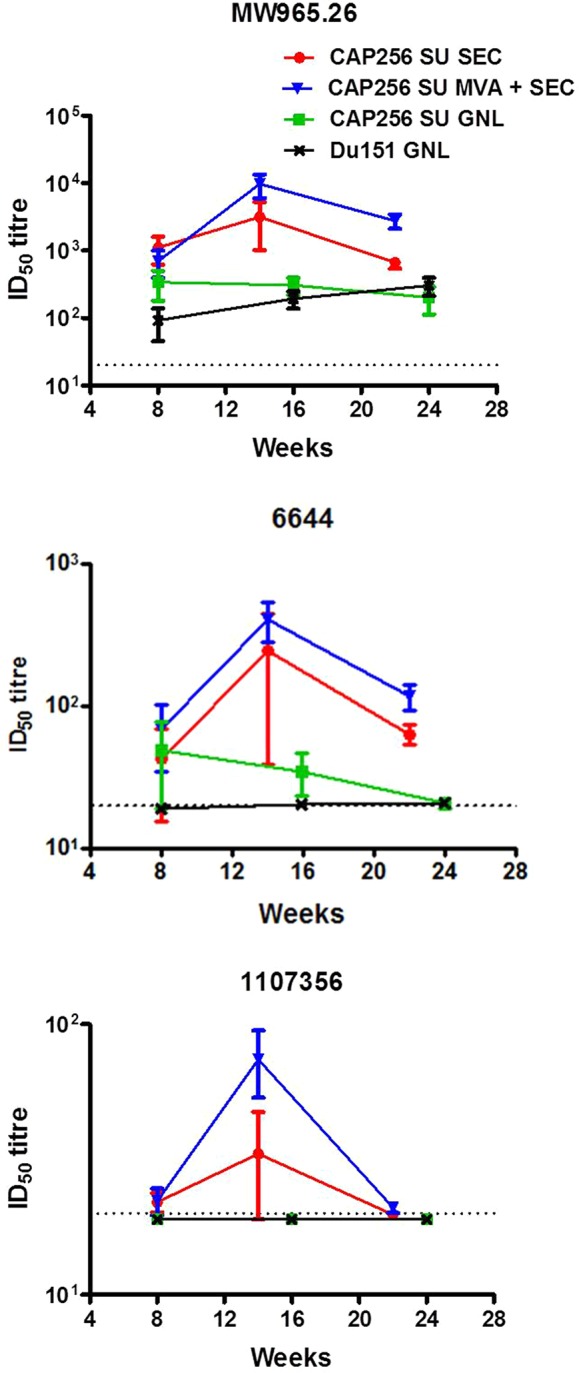
Temporal neutralizing antibodies elicited by the different vaccination regimens over the course of the experiment. The neutralizing antibody titres (ID_50_) are reflected as the mean of the reciprocal dilution required to inhibit viral entry into a reporter cell line. The dotted line at 20 represents the threshold below which neutralization activity is considered background.

Serum samples from immunized animals were assessed for neutralization activity against Env-pseudotyped viruses representing a range of neutralization sensitivities (**Tables S2A, B**) (Seaman et al., 2010). Animals immunized with SEC-purified protein exhibited a trend towards higher neutralizing titres when compared to animals immunized with the affinity-purified protein (**Figure 3**). Unfortunately, due to differences in the time points at which the assays were conducted, this could not be compared statistically.

All groups of animals developed neutralizing antibodies against the HIV-1 subtype C Tier 1A virus (MW965.26), although in the two groups immunized with the SEC-purified Env titres of over 1,000 were observed in some animals. The group primed with rMVA was determined to have significantly higher levels of neutralizing antibodies against this virus at week 22 compared to the group immunized with the SEC-purified protein only (two-tailed Mann-Whitney unpaired test, p < 0.05). Sporadic neutralization was observed against 6,644 (Tier 1B) in animals immunized with the affinity-purified protein whereas all animals immunized with the SEC-purified Env developed consistent neutralizing antibodies against this virus. Waning neutralization against 1,107,356 was also observed in animals inoculated with the SEC-purified protein. In contrast animals immunized with the affinity-purified protein did not develop any detectable neutralizing antibodies against this virus. Interestingly, more animals from the rMVA prime-SEC protein boost group (5/5) developed neutralizing antibodies against this virus than animals immunized with the SEC-purified Env only (2/5). None of the animals developed neutralizing antibodies against the autologous Tier 2 virus (CAP256 SU and Du151) (**Tables S2A, B**). Peak neutralizing antibody titres were observed after the 3^rd^ immunization and decayed to varying extents by the final bleed.

## Discussion

In view of the sequence diversity and rapid mutation rate of HIV-1, the implementation of successful HIV vaccines will require rapid production scalability, and subunit vaccines especially may be a challenge to produce in sufficient quantities. This will be particularly problematic in the developing countries that are often disproportionately affected by the pandemic, and which mostly lack the infrastructure necessary for manufacturing their own vaccines (Shao and Williamson, 2012). Given the reduced infrastructure requirements and the potential for rapid scalability of plant production systems for recombinant proteins, we have investigated the feasibility of a transient expression platform in *N. benthamiana* plants for the production of a HIV Env-derived candidate vaccine antigen.

We designed two soluble HIV Env mimetics based on rationally selected viral isolates. The antigens were designed with a flexible linker peptide at the interface of gp120 and gp41 to circumvent the need for furin-mediated cleavage, which does not occur naturally in plants (Wilbers et al., 2016). This approach is currently under investigation by a number of groups attempting to produce native-like Env trimer immunogens in mammalian cells (Kovacs et al., 2014; Georgiev et al., 2015; Sharma et al., 2015). Both antigens yielded detectable levels of expression in crude leaf homogenates. The apparent peak in protein expression occurred 5 days post agroinfiltration and western blotting of crude extract separated using denaturing SDS-PAGE yielded a band of approximately 130 kDa, as well as higher molecular weight products that were poorly resolved by electrophoresis. LC-MS analysis of gel-purified bands confirmed the identity of the HIV Env protein and verified that the unresolved higher molecular weight products observed were also HIV Env. Rosenberg and colleagues observed similar unresolved products for their plant-produced HIV Env gp140 ∆CFI antigen, although they concluded that these corresponded to oligomeric Env species (Rosenberg et al., 2013). The slight disparity in the size of our recombinant Env antigens compared to the equivalent proteins produced in mammalian cells may be a reflection of lower levels of glycan site occupancy that has been reported for other heterologous proteins produced in plants (Sharma et al., 2015).

The expression of both antigens resulted in pathology to the host plants and the pathology observed was more severe following expression of Du151 gp140 than for CAP256 SU gp140. Infiltration with recombinant *A. tumefaciens* containing an empty pEAQ-*HT* expression vector produced no obvious effects, suggesting that the pathology was a direct result of overexpressing the glycoproteins *in planta*. It is conceivable that expression of the target proteins may have exceeded the plant’s capacity to accommodate folding resulting in the accumulation of misfolded Env and the associated ER-stress response (Howell 2013). The phenotype observed in this study is consistent with considerable ER-stress and the degradation of misfolded Env proteins, as part of the ER quality control system, which would account for the low yields observed in this study (Hamorsky et al., 2015). This is currently under investigation and approaches are being investigated to improve Env production in plants and reduce the associated levels of ER-stress.

The scalability of the system was demonstrated, and a successful purification strategy was devised for the recovery of the antigens after large scale expression. The glycoproteins were efficiently bound by *G. nivalis* lectin during affinity chromatography, enabling the recovery of milligrams of protein. The inclusion of a subsequent SEC step enabled the removal of aggregated and monomeric Env species resulting in the recovery of presumably trimeric CAP256 SU gp140 antigen, although further work is required to determine if the protein is “native-like” in conformation. This could be achieved by determining the reactivity of the purified antigens with monoclonal antibodies derived from human donors and visualization of the antigens by negative stain electron microscopy (Sanders et al., 2013). The properly folded form of the protein is expected to assume a compact propeller-like conformation that exposes epitopes that are targeted by broadly neutralizing antibodies and occludes epitopes that are recognized by non-neutralizing antibodies (Sanders and Moore, 2017).

The yields of both antigens were lower than desirable, with approximately 6 mg/kg fresh weight and 5 mg/kg fresh weight of purified protein recovered by affinity chromatography for the CAP256 SU gp140 and Du151 gp140 antigens, respectively. The implementation of a subsequent SEC step resulted in a recovery of <2 mg/kg of CAP256 SU gp140. The only other report in which HIV-1 gp140 antigens were expressed in plants described raw yields of ~80 mg/kg: this was not of a purified form, however (Rosenberg et al., 2013). It is noteworthy that different HIV isolates exhibit varying levels of Env expression in mammalian cell culture-based expression systems and vary in their propensity to form trimers (Bricault et al., 2015; Julien et al., 2015; Zambonelli et al., 2016). Although other viral isolates may potentially yield higher expression levels, a strong rationale underpinned the selection of these isolates for immunogenicity studies.

Rabbits were immunized four times with the recombinant antigens to evaluate the impact of removing both aggregated and monomeric Env species, and the influence of priming the immune response with rMVA encoding Gag and a cognate Env gp150 antigen. Although this study was aimed at eliciting neutralizing antibodies against the Env glycoprotein, the inclusion of Gag could also contribute to a combination vaccine by eliciting a polyfunctional T cell response capable of suppressing viraemia and preventing disease progression following infection. The Gag used here is particularly promising as it has been optimized *in silico* to improve the coverage of common CD4^+^ and CD8^+^ T cell epitopes (Fischer et al., 2007). The co-expression of Gag and Env gp150 is also expected to result in the presentation of the Env glycoprotein on the surface of Gag VLPs when co-expressed from MVA following immunization: our group has previously reported that this Gag mosaic antigen forms VLPs *in vitro* after transfection of cells with DNA or infection of cells with MVA vaccines encoding the protein (Jongwe et al., 2016). It has been argued that presenting Env antigens on the surface of VLPs may stabilize the protein in its natural lipid membrane context, thereby presenting the antigen to the immune system in a more authentic way than in the case of soluble protein (Crooks et al., 2011; Tong et al., 2014; Crooks et al., 2015; Crooks et al., 2017). The size of VLPs also promotes entry into the lymphatic system, enabling increased interaction with professional antigen presenting cells (Tong et al., 2012; Zabel et al., 2013). In contrast, small soluble proteins are poorly taken up by the lymphatic system and are therefore comparatively less immunogenic (Bachmann and Jennings, 2010; Zabel et al., 2013). Lastly, repeating arrays of antigen on the surface of VLPs enable cross-linking of B cell receptors, resulting in the induction of long-lived antibody responses (Bachmann et al., 1993; Bachmann and Jennings, 2010).

The Env produced in plants was well tolerated in rabbits and demonstrated promising immunogenicity. Binding antibodies were detected after the first immunization and increased in titre over the course of the experiment. The boosting effect became less pronounced after the first two immunizations and binding antibody levels were observed to decline to some extent over time. Similar levels of binding antibodies against the matched antigen were observed in all groups with no statistically significant differences observed. However, animals immunized with the SEC-purified protein developed significantly higher levels of binding antibodies after the 3^rd^ and 4^th^ immunization than animals immunized with the protein that had only been lectin affinity-purified. Encouragingly, all immunization regimens elicited binding antibodies directed at the V1V2 loop, which was shown to be a correlate of vaccine-mediated protection in the RV144 trial (Haynes et al., 2012). Furthermore, animals immunized with the SEC-purified protein exhibited a distinct trend towards higher neutralization titres against several Tier 1 viruses. It is assumed that contaminating aggregates and monomeric Env species present in the affinity-purified vaccines elicited “off-target” antibodies that could not engage the native Env glycoprotein, resulting in lower levels of neutralizing antibodies. Interestingly, peak neutralizing antibody titres in both groups were observed after the 3^rd^ immunization, and subsequently declined despite an additional inoculation.

Priming rabbits with rMVA followed by boosting with the sec-purified protein further improved the induction of neutralizing antibodies. Although this difference was only significant for MW965.26 at week 22, a trend was seen across the different pseudoviruses that were tested. Despite MVA not being replication-competent, its ability to infect cells and express heterologous proteins *in vivo* will lead to processing of the antigens *via* the intracellular proteasome and cross-presentation by MHC receptors, resulting in the induction of both CD4^+^ and CD8^+^cells (Sutter and Moss 1992). The induction of improved CD4^+^ T cell help may have improved the elicitation of neutralizing antibodies following immunization with the SEC-purified protein (Crooks et al., 2007). In further support of this heterologous prime-boost approach, the only clinical trial to report efficacy against HIV acquisition employed a canary pox prime followed by a recombinant Env protein boost (Rerks-Ngarm et al., 2009; Robb et al., 2012).

This is to our knowledge the first study to report the successful production of apparently trimeric soluble HIV-1 Env protein, and to investigate its immunogenicity, and represents an important first step in the development of a candidate plant-produced HIV vaccine for clinical trial. Further work is ongoing to address limitations of yield and potential plant-specific differences in glycosylation and folding in order to improve the induction of neutralizing antibodies. The work described here highlights the importance of stringent purification of Env glycoproteins for use as vaccine immunogens and the benefit of priming with a recombinant poxvirus to improve humoral responses against the Env glycoprotein.

## Data Availability Statement

All datasets generated for this study are included in the article/**Supplementary Material**.

## Ethics Statement

The animal study was reviewed and approved by University of Cape Town Health Sciences Faculty Animal Ethics Committee (AEC 014-30 and AEC 015-05).

## Author Contributions

EM conducted the plant expression, purification, and immunogenicity experiments and compiled the manuscript. RC designed the sequences of the HIV antigens and cloning strategies used to construct the plant and mammalian expression vectors and MVA. RC, AM, ER, and A-LW supervised the experimental work and contributed to experiment design. MD and PX produced the mammalian cell-derived Env protein and contributed to experiment design. BW assisted with the development of a purification strategy and assisted with protein purification experiments. TH performed and CC aided with the neutralization assays. LM supervised neutralization assay experiments and contributed to interpretation of the results. All authors provided feedback on the manuscript.

## Funding

This work is based upon research supported by the South African Medical Research Council with funds received from the South African Department of Science and Technology and the South African Research Chairs Initiative of the Department of Science and Technology and National Research Foundation.

## Conflict of Interest

The authors declare that the research was conducted in the absence of any commercial or financial relationships that could be construed as a potential conflict of interest.
