# Supplementary figures and images for: Production and Immunogenicity of Soluble Plant-Produced HIV-1 Subtype C Envelope gp140 Immunogens
Source: Front Plant Sci. 2019 Oct 30;10:1378. doi: 10.3389/fpls.2019.01378 (PMC6831737; doi:10.3389/fpls.2019.01378)

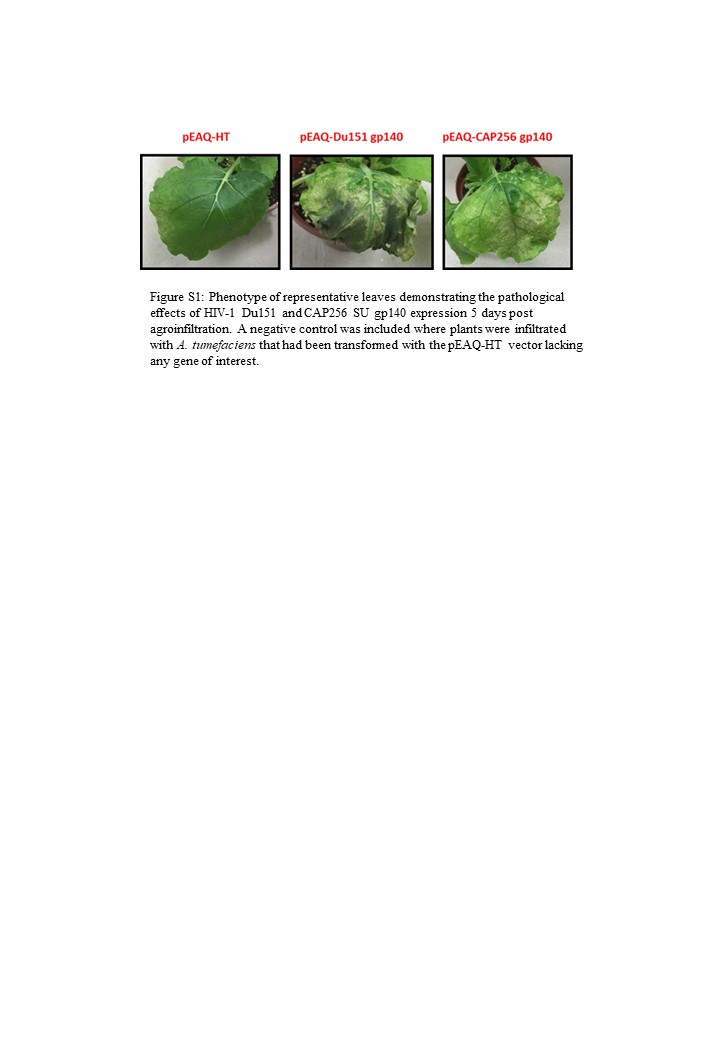

Supplement: Supplementary file 1 [file Image_1.jpeg]

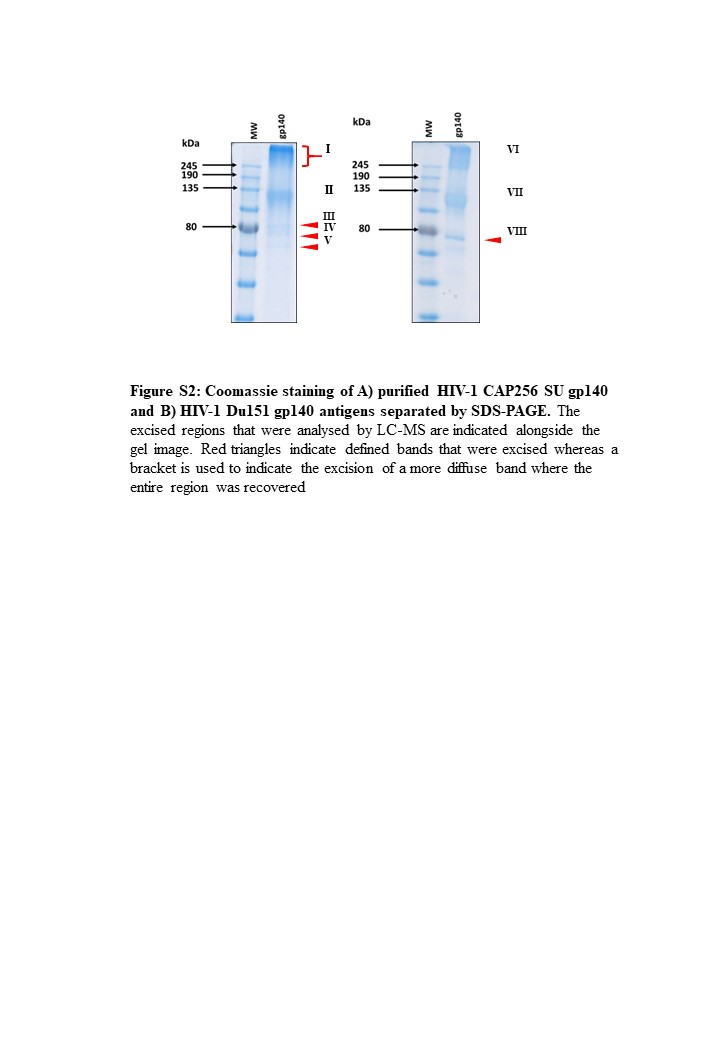

Supplement: Supplementary file 2 [file Image_2.jpeg]
