# Supplementary material for: Production and Immunogenicity of Soluble Plant-Produced HIV-1 Subtype C Envelope gp140 Immunogens
Source: Front Plant Sci. 2019 Oct 30;10:1378. doi: 10.3389/fpls.2019.01378 (PMC6831737; doi:10.3389/fpls.2019.01378)
Supplement: Supplementary file 3 [file Table_1.docx]

**Table S1: LC-MS analysis of Coomassie stained protein bands recovered from SDS-PAGE gels.** The protein identity reflects the major protein hit of each band. (FDR = false discovery rate)

| **Band** | **Protein identity** | **Log P** | **FDR (%)** |
| --- | --- | --- | --- |
| **I** | HIV-1 Env glycoprotein gp160 (isolate 92BR025) | 28.30 | 1.5 |
| **II** | HIV-1 Env glycoprotein gp160 (isolate 93BR020) | 33.88 | 2 |
| **III** | Chaperone protein DnaK (*A.tumefaciens fabrum*) | 8.58 | 2.8 |
| **IV** | Chaperone protein DnaK (*A.tumefaciens fabrum*) | 13.80 | 2.5 |
| **V** | Chaperone protein DnaK (*A.tumefaciens fabrum*) | 7.65 | 1.8 |
| **VI** | HIV-1 Env glycoprotein gp160 (isolate 93BR020) | 117.67 | 2.1 |
| **VII** | HIV-1 Env glycoprotein gp160 (isolate 93BR020) | 67.48 | 0.5 |
| **VIII** | Luminal-binding protein 5 | 201.35 | 0.5 |
