# Supplementary material for: Production and Immunogenicity of Soluble Plant-Produced HIV-1 Subtype C Envelope gp140 Immunogens
Source: Front Plant Sci. 2019 Oct 30;10:1378. doi: 10.3389/fpls.2019.01378 (PMC6831737; doi:10.3389/fpls.2019.01378)
Supplement: Supplementary file 4 [file Table_2.docx]

**Table S2: Serum ID50 neutralizing antibody titres elicited by different vaccination regimens.** (A) Animals were immunized at weeks 0, 4, 12 and 20 with 50 μg of *Galanthus nivalis* lectin-affinity purified CAP256 SU (CAP256 SU GNL) or Du151 gp140 protein (Du151 GNL). (B) Animals were immunized with 50 μg of SEC-purifiedCAP256 SU gp140 protein at weeks 0, 4 and 12 (CAP256 SU SEC), or with 1×10^8^ pfu MVA at weeks 0 and 4, followed by 50 μg of SEC-purified CAP256 SU gp140 protein at weeks 12 and 20 (CAP256 SU MVA+SEC). Sera from immunized animals were assessed for neutralizing activity against a panel of Env-pseudotyped virions over the course of the experiment. Neutralization of each pseudovirus is presented as the serum dilution required for a 50% reduction in entry of the infecting virus into a reporter cell line (ID50). In both tables the pseudovirus tested for neutralization is indicated with its tiered neutralization sensitivity phenotype. (WK= week)

A

B

| **Serum ID_50_** | |
| --- | --- |
|  | <20 |
|  | 20 - 100 |
|  | 100 - 1000 |
|  | > 1000 |
